# Advanced Handover Decision with Mobility Awareness for 5G/6G Mobile Networks in Ultra-Dense and Smart Cities Area

**DOI:** 10.3390/s26144374

**Published:** 2026-07-10

**Authors:** Soule Issa Loutfi, Ibraheem Shayea, Ufuk Tureli, Waheeb Tashan, Laura Aldasheva, Akzhibek Amirova, Didar Yedilkhan, Saule Amanzholova

**Affiliations:** 1 Electronics and Communication Engineering Department, Faculty of Electrical and Electronics Engineering, Yildiz Technical University, 34220 Istanbul, Turkey; loutfi.issa@std.yildiz.edu.tr (S.I.L.); utureli@yildiz.edu.tr (U.T.); 2 Electronics and Communication Engineering Department, Faculty of Electrical and Electronics Engineering, Istanbul Technical University, 34467 Istanbul, Turkey; 3 Department of Electronics and Communication Engineering, Kocaeli University, 41001 Kocaeli, Turkey; waheeb.tashan@kocaeli.edu.tr; 4 School of Cybersecurity, Astana IT University, Astana 010000, Kazakhstan; laura.aldasheva@astanait.edu.kz (L.A.); akzhibek.amirova@astanait.edu.kz (A.A.); s.amanzholova@astanait.edu.kz (S.A.); 5 Smart City Research Center, Astana IT University, Astana 010000, Kazakhstan; d.yedilkhan@astanait.edu.kz

**Keywords:** 6G networks, handover decision, mobility awareness, mobility management, ultra-dense and smart cities

## Abstract

Fifth-generation advanced (5G-A) and sixth-generation (6G) networks in ultra-dense and smart cities offer an efficient solution for addressing the growing number of mobile users while ensuring high throughput and full coverage. However, the handover decision (HOD) remains a critical challenge in wireless networks, especially for high-speed users in millimeter-wave (mmWave) communication environments. The present paper proposes a mobility-awareness HOD algorithm that combines two decision algorithms: reference signal received power (RSRP) and signal-to-interference-plus-noise ratio (SINR). The algorithm makes decisions based on real-time user experiences collected every 40 milliseconds during the user’s mobility. The research was conducted using MATLAB 2023a and advanced 5G and 6G tools. All system settings, functions, mobility models, and simulation criteria were defined based on 3GPP specifications. The algorithm guarantees high-quality connectivity and smooth user transitions across 5G and 6G networks under diverse mobility conditions. The results show that Systems 2 and 4 minimize 96.97% and 99.99% of handover ping-pong (HOPP) compared with Systems 1 and 3. The simulation results reveal that the proposed algorithms in Systems 2 and 4 provide noticeable enhancements in network performance, with radio quality metrics of 16.32% and 8.04% for RSRP, 168.99% and 407.31% for SINR levels, and 55.11% and 16.04% improvements in throughput across various mobile speed scenarios compared with Systems 1 and 3.

## 1. Introduction

The ongoing advancement of wireless communication technology has led to the development of 6G networks that offer ultra-reliable, high-speed, and low-latency connectivity. The future generation of networks in smart cities is likely to change the digital landscape, affecting various spheres of modern life, including healthcare, autonomous vehicles, the Internet of Things (IoT), and industrial control systems. 6G networks are expected to provide unprecedented connectivity due to the combination of terrestrial and non-terrestrial networks, the introduction of intelligent optimization solutions for the network, and the opportunity to process essential real-time information [1,2]. Even though 5G networks are already incorporating artificial intelligence (AI) and machine learning (ML) to optimize the network, 6G is expected to go a step further by incorporating these technologies as part of autonomous, real-time, and context-aware network management. Moreover, these networks will use advanced handover decision-making algorithms to maintain seamless connections in extremely dynamic, heterogeneous environments [3]. Handover management is an intrinsic component of 6G networks in ultra-dense environments, and it is crucial for ensuring uninterrupted communication, as mobile users are aware of network nodes. In conventional cellular networks, HO decisions are mostly determined by signal strength criteria. However, such solutions often lead to unnecessary handovers, exacerbating network congestion and degrading the overall quality of service (QoS). The deployment of ultra-dense tiny cells, the integration of terrestrial and non-terrestrial networks, and enhanced user mobility complicate HO decision-making in 6G networks. Consequently, effective HO management is essential for minimizing latency, optimizing network resource allocation, and ensuring continuous service delivery, especially in smart city contexts where real-time communication and automation are vital [4,5,6].

Meanwhile, HetNets with a large number of connected devices pose significant mobility management challenges due to the high frequency of HOs and the large number of connected devices, which together result in massive HOs and network traffic. These difficulties include HOP, HOPP, radio link failure (RLF), and data rate (throughput). Furthermore, maintaining high-quality connectivity during enhanced-speed scenarios remains a significant challenge in mobility management, as high-speed rail systems and unmanned aerial vehicles (UAVs) can trigger frequent handovers [7].

Mobility awareness HO is vital for efficient inter-cell resource allocation by predefining handover control parameters (HCPs), especially the handover margin (HOM) and the time to trigger (TTT). For fixed HCP systems, these parameters affect network stability, service reliability, and the distribution of user data rates. However, improper values can degrade network performance. A high HOM delays HO, keeping the user attached to the serving cell even as the conditions deteriorate, and it may result in poor radio conditions after HO. Controversy: Low HOM may fail to offload traffic from overloaded cells, leading to poor throughput and service disruption. Likewise, a short TTT reduces HO delay and can improve throughput, but it can also increase the probability of HO ping-pong. A long TTT can cause handover HO, leaving the user in a congested cell for longer periods and thus compromising the overall network performance [8].

The HO decision is vital and needs to be executed efficiently in high-speed mobile networks to ensure continuity of communication as a user moves within the range of a cellular system, which consists of many base stations (gNBs) [9]. The basis of these evaluations is largely grounded in major elements, including HOM and TTT. There are two key HO decision metrics that quantify the RSRP and SINR of the serving and target gNBs. They show the signal level and quality, making it easier to select the target gNB for handover. To initiate an HO decision, the RSRP and SINR of a target gNB must exceed both the HOM and the RSRP or SINR, respectively, of the serving gNB. Therefore, 5G/6G network mechanisms in the HO decision can rely on these factors. These factors are constantly evaluated by the system, and a handover is performed when the user equipment needs to connect to the best gNB [10].

Various mobility management algorithms for HO decision-making beyond 5G and 6G networks have been widely discussed in the literature. An HO decision algorithm based on Bayesian regression was suggested in [11] for LTE-R networks, where it is assumed that the HO conditions are independent. The algorithm estimates the crossing time for the cell boundaries and decides when to start the HO process (with a fixed preparation delay). This assumption is not practical, as it fails to consider the influence of many dynamic factors on HO performance. In [12], the focus on the HO decision process in LTE-R systems is placed on the A3 event to enable efficient HO in high-speed trains. The traditional fixed-threshold approach is not suitable for variable train speeds and environments and is susceptible to handover failures and ping-pong effects. To address this, an adaptive HO algorithm based on elliptic functions adjusts the hysteresis threshold according to train speed and position, improving HO timing and success. The method is effective, but it still needs to be tuned and optimized for a complex environment [13].

Most of these algorithms improve HO performance; they lack robustness and are not optimal for determining suitable HCP values in new-generation networks, such as 5G/6G. The main reason is that fourth-generation technologies show that the design systems of these algorithms differ in their specifications and requirements from those of 5G and 6G networks. Although the algorithms tailored for preceding cellular networks are inadequate to meet the demand of future network deployment [14]. Consequently, additional research encompassing a wide range of mobility and deployment scenarios is necessary for the 5G network. All aforementioned methods were developed to operate within a core optimization model.

The proposed method is based on integrating dual-condition HO decision-making with mobility awareness for 5G advanced and 6G networks. However, traditional HO depends on a single RSRP and SINR measurement. The proposed method evaluates the high-signal and minimum-threshold conditions before HO execution. In addition, the HO decision for the mobility condition reduces significant HO ping-pong and unnecessary HO while maintaining seamless connectivity and QoS and improving throughput across various mobile-speed scenarios in ultra-dense 5G/6G networks.

The main contributions of this paper are summarized as follows:

- Four different systems (i.e., System 1, System 2, System 3, and System 4) are presented with identical configurations of HCPs across multi-tier networks and are examined at all mobile speeds (up to 140 km/h) to demonstrate their performance in each speed condition.
- A two-step method is proposed to evaluate the target and serving cell for the SINR-based HO decision algorithm for Network 1 and Network 2. The HO decision algorithm is designed to regulate the HO process between the serving and target gNBs.
- The system’s performance has been illustrated using various KPIs, including RSRP, SINR, HOP, HOPP, RLF, and data rate. Investigating four systems across multiple mobile-speed scenarios will shed light on how HCP configurations affect system performance under varying speeds.

The rest of this paper is composed as follows. In Section 2, we present the research background, introduce related studies, and elaborate on the study challenges and limitations. In Section 3, the implementation of two systems and the HO decision algorithm is detailed. In Section 4, the system model is presented. In Section 5, KPI’s formulation and analysis have been developed. The simulation results and discussion are presented in Section 6. Finally, the conclusions are discussed in Section 7.

## 2. Background and Related Studies

This section outlines the research background and related studies, mostly addressing HOD with mobility awareness in 5G/6G networks. The first part examines the study background, and the following subsection provides a concise overview of several research publications.

### 2.1. Research Background

The emergence of mobile network generations is likely to enable a wider range of services for mobile users. This diversification of services presents various context-specific standards. Quality-of-service measurement techniques have been developed to control the quality level between mobile users and networks. Introducing HOD to wireless networks is associated with the QoS delivered by the network and the QoS experienced by the end user. The performance, or key performance indicator, handover measures the parameters of link quality, including RSRP and SINR, to determine the best target radio link access. The decision on the ideal radio access for the target in wireless networks is a complex process that depends on a myriad of factors. In accordance with this assumption, we consider the mobility awareness of information within the environment that describes the condition of a given user and network, including location, time, and the activities of the user and the network operator. In this section, the author provides a brief description of 6G networks and the history of HOD and mobility awareness in HO decisions.

#### 2.1.1. 6G Networks

6G wireless systems are the most modern development in cellular technology and have progressed to provide numerous connectivity opportunities at breakneck speeds measured in microseconds. The next-generation network remains an open research challenge based on high radio frequencies and greater capacity, with a latency 1000 times higher than that of 5G. As 5G is implemented, research institutes around the globe are now working on 6G, which is estimated to be commercially deployed in 2030 [15,16,17]. The 6G network is the next major advancement in wireless communication, building on 5G with ultra-high frequencies to support much higher data rates. The integration of AI and ML is anticipated to revolutionize mobile network technology by enhancing efficiency and performance. 6G will offer higher connectivity speeds, enhanced security measures, and reduced latency compared to 5G. It will be the fastest generation of wireless communication, with speeds expected to range from 1 to 10 terabits per second.

With the development of 6G technology, digital communication is expected to experience major improvements in speed, security, and service quality. Designed to revolutionize connectivity, 6G is expected to deliver up to 10 million devices per km^2^ and 1 Gb/s per square meter of traffic density. Based on its key performance indicators (KPIs), 6G is expected to achieve 99.9% coverage and 99.9999% reliability. Its prime objective is to satisfy the rapidly increasing requirements of future wireless networks, where peak data rates can reach values equal to or higher than 1 Tbps, which is almost 100 times faster than current 5G networks. Individual user data rates are also projected to be as high or higher than 1 Gbps. Further, it is expected that 6G will be an extremely advanced wireless communication technology, with energy efficiency that is 10 to 100 times greater than 5G and spectrum efficiency that is 5 to 10 times greater [18,19].

However, the most radical aspect of 6G is its multisensory nature of architecture. In contrast to its forerunners, 6G is expected to provide unlimited wireless connectivity and to combine a diverse set of functions, including communication, metering, data storage, computation, control systems, radar, imaging, and navigation into a single system. This convergence of varied technological capabilities is likely to unlock new possibilities never before seen, creating a more connected and more technologically intense future.

#### 2.1.2. Mobility Awareness in HO Decision

**Figure 1:**
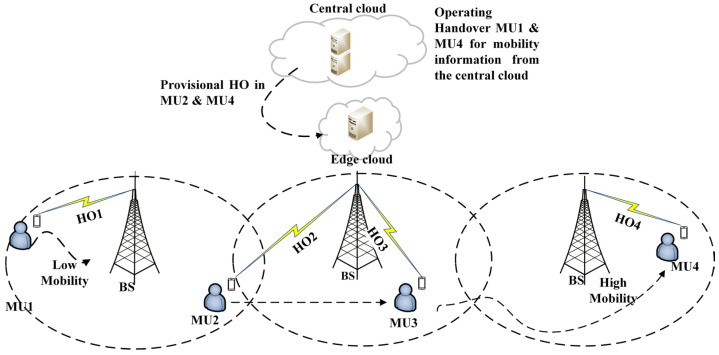
Mobility awareness within the handover decision.

Mobility awareness in HOD is an essential feature of 6G networks. Since 6G is designed to offer ubiquitous, uninterrupted connectivity, higher data rates, reduced latency, and more reliable connections, the ability to control HO efficiency becomes an even greater concern. Figure 1 represents the mobility-awareness systems in HO decisions. This system comprises N mobile users (MUs). Based on advanced wireless technologies, the network provider will establish a network function for HO in edge computing and artificial intelligence to support mobility management. The integration of these technologies in the 6G network brings more significant improvements in the QoS and efficiency of MUs [20]. However, mobility awareness is integral to the handover decision-making process through the implementation of the mobility-awareness personalized handover function provisioning system. With its central controller, the system efficiently collects real-time mobility information for each MU and identifies MUs with high mobility that require more efficient HOs, providing a robust and reliable solution.

First, the scenario related to the HO and mobility management function to enhance the communication systems. Mobility information manages essential data to identify the location of the MU and QoS and to improve responsiveness to the user’s mobility awareness. However, the mobility configuration function updates the HO to determine the location area of the MU within the network operators. The communication functionality, with its seamless signaling, enables signaling from the horizontal direction between the transmitter and receiver in one cell to another base station, ensuring smooth and uninterrupted communication.

Therefore, the performance metrics, such as average signaling overhead and edge-cloud resource usage, highlight the advantages of this mobility-awareness approach over traditional handover management systems, ultimately enhancing the efficiency of mobility management in 6G networks.

#### 2.1.3. Handover Decision in 6G Networks

**Figure 2:**
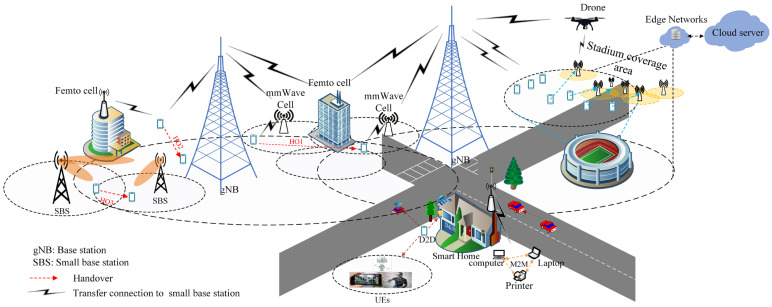
Handover concept for a 6G cellular network environment in ultra-dense settings.

The handover decision in 6G technologies is not only an issue but also a key consideration requiring extensive research in wireless networks. This choice will provide a fully smooth interconnection and maximum performance with advanced telecommunications networks [21]. The design and development of 6G network systems involve a broad range of important considerations and design requirements in ultra-dense environments, underscoring the importance of current research on wireless networks. The highest-frequency bands, especially in the terahertz (THz) and sub-terahertz (sub-THz) ranges, are among the most distinguishing features of 6G compared with its preceding generations. Although these frequency bands offer significant benefits in terms of bandwidth and network capacity, they also present significant technical problems in the form of severe signal attenuation on the one hand and vulnerability to physical barriers and blockages on the other hand [22,23]. The architecture of 6G networks is likely to be based on the massive deployment of ultra-dense networks (UDNs), characterized by multiple clusters of small cells across varied geographic locations. It is a next-generation architecture designed to support a cellular network environment in which several small-cell networks can operate simultaneously within the coverage area of a single base station. Such tightly spaced small-cell networks are becoming more and more in demand, as shown in Figure 2 [24], especially due to the large number of HO events recorded as users move in/out of various smart city areas. A high-quality, seamless HOD mechanism will be imperative to sustaining and improving QoS between users and base stations (BSs). Moreover, Figure 2 outlines the future outlook for wireless networks in ultra-dense deployment settings, illustrating that it is possible to achieve high network capacity, increased data throughput, and greater connectivity range between edge networks and cloud servers.

It is expected that the next generation of network infrastructure will utilize the simultaneous capacity of cellular network signals and high-power base station towers while maintaining cost-effectiveness and sufficient power efficiency required to implement 5G and 6G networks and guaranteeing a decent QoE. However, the shift to 6G is also associated with a new range of issues and potential, the most notable being the threat of higher inter-cell interference, a significant increase in the number of handover events, and increased power consumption [22,25]. The high number of mobile network device users and the rise of many more advanced Internet of Things (IoT) applications have helped to drive significant growth in the amount of data traffic across communication systems. To address these expanding requirements, 6G is proposed to support a new generation of applications that demand extreme reliability, ultra-low latency, and record data rates, including but not limited to holographic communication, tactile internet, and support for fully autonomous vehicular systems [22,26]. Our expertise is clearly needed to navigate these challenges and seize these opportunities.

**Figure 3:**
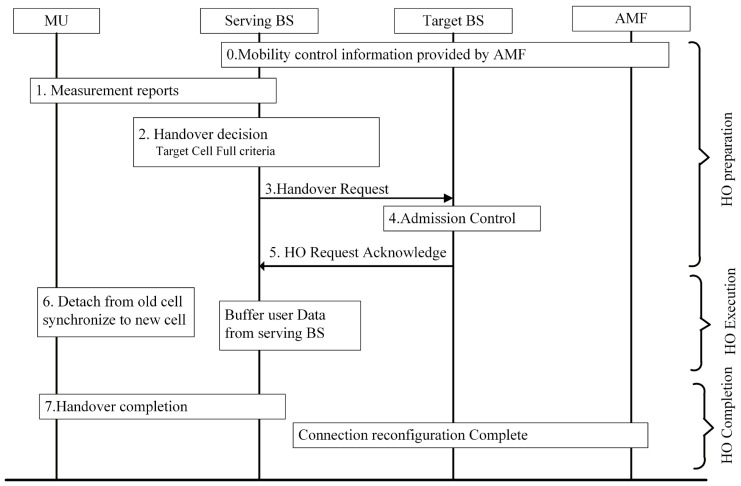
HO decision within the HO procedure.

Handover is a critical process that facilitates the transfer of communication between base stations (BSs) to enhance service quality for MUs in cellular networks. The process is initiated by the mobile station (MS), which periodically measures the RSRP of available BSs and transmits measurement reports (MRs) to the serving BS. Using these reports, the serving BS decides which target cell to communicate with to maintain continuous and reliable communication. Figure 3 presents an illustration of the HO process, a crucial stage in which the HO decision is made, influencing the entire process [27]. This scenario illustrates the wireless network control handover (CHO) process in B5G during the initialization of a 6G network. The CHO introduces features in 3GPP Release 17 that enhance HO reliability and enable mobility management across both terrestrial and non-terrestrial networks.

HO preparation is divided into five phases. Step 1: The serving BS sets the MS measurement procedures, and the MS transmits MRs to the serving BS based on the measurement settings. Step 2: The serving BS makes an HO decision to identify the optimal target based on the information received in the form of MRs and radio resource management (RRM). Step 3: The serving BS sends an HO Request message to the target BS, and the necessary information is contained in a transparent RRC container. Step 4: Admission Control is performed by the target BS.

Step 5: The target BS is ready for the handover, including layers 1 and 2, and sends the HO Request Acknowledge to the serving BS, such as a transparent container as an RRC message, to complete the handover.

In HO execution, HO is performed immediately upon reception of the HO command. In Release 16, 3GPP introduced the conditional handover (CHO) feature, which allows the target BS(s) to provide execution conditions in the CHO configuration to the UE. Handover is performed (HO execution initiation) when the CHO conditions are met.

In HO completion, the MS synchronizes with the target BS, and the reconfiguration complete (RRC) message is sent to the target BS to complete the RRC handover procedure. The MS does not part ways with the serving BS upon receiving the RRC Reconfiguration message unless the target BS sends an HO success message to the serving BS confirming that the MS has reached the target cell successfully. Lastly, the serving BS transmits the SN status transfer message and begins normal forwarding of the data.

### 2.2. Related Studies

For efficient mobility and HO management, there are related studies in the literature presented in Table 1 [28,29,30,31,32,33,34,35,36,37]. These works can be categorized as architecture- and fuzzy-logic-based HO, optimization for multi-attribute decision-making (MADM)-based HO, adaptive HO/TTT algorithms [28,29,30,31,32], and machine learning-based HO and reinforcement learning (RL)-based HO [33,34,35,36,37].

In [30], HO is considered a key challenge in dense HetNets. The study illustrates an efficient DL algorithm to decrease energy consumption due to the transmission and HO processes. The proposed approach, in contrast to traditional HO methods that focus primarily on RSRP and fixed-energy models, enhances decision-making and provides 10% to 30% energy savings over traditional approaches. It is also more practical than Markov-based methods in dynamic network environments.

Satapathy et al. [31] introduced a novel multicriteria-based vertical handover (VHO) decision algorithm to ensure seamless connectivity and maintain QoS in HetNets during network handover. The technique uses the fuzzy analytic hierarchy process (FAHP) and the technique for order of preference by similarity to ideal solution (TOPSIS) to evaluate network-, terminal-, and service-related criteria. The algorithm accounts for dynamic base-station characteristics and user preferences, reducing HO failures, delays, and energy consumption while enhancing the user experience. The proposed context-aware approach differs from traditional single-criterion approaches, which primarily depend on signal strength and aim to improve service continuity and resource utilization in complex network environments.

In 2023, Satapathy et al. [32] presents an adaptive context-aware vertical HO decision algorithm for HetNets. The algorithm applies contextual information, e.g., user conditions, network characteristics, and application needs, to enhance the handover decision. It aims to ensure smooth connectivity while minimizing call blocking, handover time, and energy usage. The method also reduces packet loss and unneeded handovers, particularly in high-mobility environments, thereby enhancing overall network performance and user experience.

In [33], a mobility-aware handover (HO) decision framework for 5G network slicing was presented, leveraging cloud-network slicing (CNS) and multi-access edge computing (MEC) to enhance mobile health (m-health) services. The study emphasizes the need to maintain connectivity during mobility events, such as ambulance transport, when real-time data is critical for patient care. It states that common HO mechanisms, based on physical-layer parameters, are insufficient to meet the stringent service-level agreements (SLAs) of e-health applications. As a result, a proactive, automated mobility management system based on ML is proposed to optimize HO decision-making and resource allocation, enabling seamless service delivery in federated cloud networks.

In [34], an intelligent HO system was presented for 5G mmWave networks based on deep learning. The approach is based on double deep reinforcement learning (DDRL) and uses movement patterns of users and the network structure to make more appropriate HO decisions. It is primarily designed to minimize handover rate and maximize system throughput. The comparison results demonstrated that DDRL is superior to traditional strategies, such as rate-based handover and SMART, in terms of delay and user satisfaction for urban network environments in the simulation results.

In [35], a vertical HO decision method for vehicular networks was proposed using dynamic Q-learning and fuzzy convolutional neural networks (CNNs). The study is designed to overcome the limitations of traditional methods, such as TOPSIS, K-NN, and AHP, which are not well suited to diverse vehicle environments. The proposed solution is designed to enhance HO management to reduce HOs and HO failures by 25% and 2%, respectively, and to reduce packet loss and improve network efficiency to facilitate future IoV systems.

In [36], Kumar et al. proposed a vertical HO decision-making algorithm based on the RL and a neuro-fuzzy graph neural network in the Internet of Vehicles (IoV). The method helps mitigate issues such as HO failures and network delays, particularly for high-speed vehicles. The proposed algorithm enhances QoS and throughput using the cluster-based routing algorithm. Comparisons with existing methods indicated that the simulation results obtained by OMNeT++ were better, which implies that this simulation tool has the potential for enhancing the connectivity of intelligent transportation systems.

In [37], a reinforcement learning (RL)-based approach to the HO decision problem in ultra-dense HetNets was proposed. The method uses a deep Q-learning algorithm to improve handover strategies by optimizing multiple factors beyond RSRP, such as energy consumption and network connection time. The simulation results in MATLAB demonstrate that the proposed approach outperforms conventional approaches, particularly under quasi-static interference conditions, by learning environmental characteristics and avoiding unnecessary handovers. The study also demonstrates scalability for large networks without increased computational complexity, resulting in better network performance and user experience.

Several HO methods have been illustrated in the literature review, such as reinforcement learning, adaptive HOM/TTT, and ML-based HO, to improve mobility awareness in 5G/6G networks. However, the proposed dual-condition HO decision framework provides a lightweight and efficient HO that maintains stable mobility performance, reducing ping-pong and enhancing throughput, as presented in Section 6.

### 2.3. Challenges and Limitations of Studies

The related studies in this research highlighted several challenges and limitations in HOD systems that future networks need to address. A key issue is mobility prediction, a major challenge for current models that do not capture rapid changes in users’ movement in urban areas with varying traffic conditions. However, limited movement history makes it hard to predict users’ future locations. In addition, future research should develop advanced ML techniques that leverage real-time information to enhance mobility prediction. Addressing security and privacy concerns in mobility data collection is crucial for user trust and regulatory compliance, while integrating multi-access networks is a critical issue, as users switch between Wi-Fi, 4G LTE, and 5G. Even so, the user will require seamless HO processes to enable uninterrupted connectivity. Future networks ought to adopt integrated space–air–ground–sea systems as the key to global network coverage, high data rates, and low latency. Scalability and adaptability are also key, as HOD algorithms need to work in large, dynamic environments such as IoT and vehicular networks. Studies carried out so far tend to focus on 4G and 5G, which may be insufficient to meet the requirements of 6G. Furthermore, few of these studies have considered user experience, and security and privacy concerns in the collection of mobility data remain important future challenges.

## 3. Proposed Models

This study proposes an HO decision algorithm based on instantaneous SINR to make real-time HO decisions for users connected to 5G and 6G networks operating at 28 GHz and 120 GHz, respectively. This study also investigates HO performance based on RSRP as the benchmark for the SINR-based model. The conducted research work consists of paired systems, which are known as Systems 1 and 2 for 5G networks and Systems 3 and 4 for 6G networks. System 1 and System 2 work based on 5G specifications with operating frequency bands at 28 GHz, considering HO decisions based on RSRP and SINR, respectively. Meanwhile, System 3 and System 4 work based on 6G specifications with operating frequency bands at 120 GHz, considering handover decisions based on RSRP and SINR, respectively. The HOD algorithms are controlled by predefining fixed HCP settings for each system. Meanwhile, the proposed HO decision considers resource availability in the serving and target cells as the main factor in making the HO decision for 5G and 6G networks.

### 3.1. Implementation of the Systems Based on RSRP

**Figure 4:**
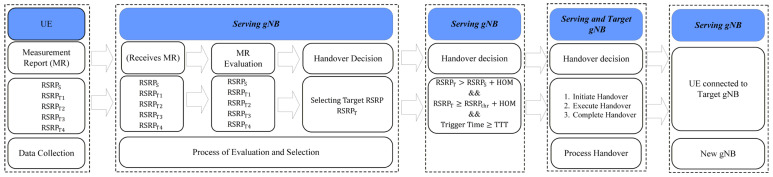
Model structure of the HOD algorithm based on RSRP functions for 5G/6G networks.

Figure 4 presents the UE-assisted, network-controlled HOD model for advanced 5G/6G networks, which employs probabilistic dual-condition algorithms to enhance the stability of seamless wireless communication and prevent unnecessary cell switching. The process commences with the UE measuring the signal quality of RSRP and reporting it to the serving base station (gNB). The decision logic lies in the HO decision block, which triggers a handover to systems (Target cell) when the conditions are met simultaneously. However, the relative conditions, such as ${RSRP}_{T}>{RSRP}_{S}\;+HOM$, indicate that the target cell must exceed the serving cell signal ${RSRP}_{T}$. By defining a fixed HOM, this ensures that the target is significantly better.

The HO decision is triggered according to the dual function simultaneously satisfied:

(1) $${RSRP}_{T}>{RSRP}_{S}+HOM\;\&\&\;{RSRP}_{T}\geq {RSRP}_{thr}+HOM$$

where ${RSRP}_{T}$ and ${RSRP}_{S}$ are the RSRP of the serving and target BSs, respectively, ${RSRP}_{thr}$ represents the RSRP threshold, and HOM represents the handover margin.

Further, the handover decision satisfies the conditions as follows:

(2) $$HOD\left(t\right)=\{\begin{array}{cc} 1 & ,if\;{RSRP}_{T}>\;{RSRP}_{S}+HOM\;and\;{RSRP}_{T}\geq {RSRP}_{thr}+HOM\\ 0 & \;\;\;\;\;otherwise \end{array}$$

where HOD(t)=1 indicates that the HO is executed, and HOD(t)=0 indicates that the UE is still connected to the serving cell.

The proposed dual-condition HO for RSRP enhances mobility management in 5G/6G networks by jointly considering relative and signal-quality measurements before triggering a handover procedure.

The first condition in Equation (1) ${RSRP}_{T}>{RSRP}_{S}+HOM$ ensures that the target BSs provide better signal strength than the serving BS. The study comparison prevents unnecessary HOs, reducing ping-pong HO. However, the second condition in Equation (1) ${RSRP}_{T}\geq {RSRP}_{thr}+HOM$ diagnoses that the target BS satisfies the minimum acceptable signal threshold for reliable communication. This condition prevents HO execution toward weak or unstable BS. The proposed dual condition improves HO stability in 5G networks and connection reliability under the high-mobility conditions expected in 6G ultra-dense networks. Compared with traditional HO approaches, the proposed dual-conditional mechanism provides many advantages to improve radio link stability, enhance data rate performance, lower HO failure probability, and deliver better quality of service. Also, the proposed HO is suitable for 6G environments with ultra-dense small cell deployment, high-frequency communication, high-speed mobility scenarios, and AI-driven adaptive network management. Therefore, the dual-condition improves HO decision accuracy while maintaining seamless connectivity and efficient utilization in future 6G networks.

### 3.2. Implementation of Systems Based on SINR

**Figure 5:**
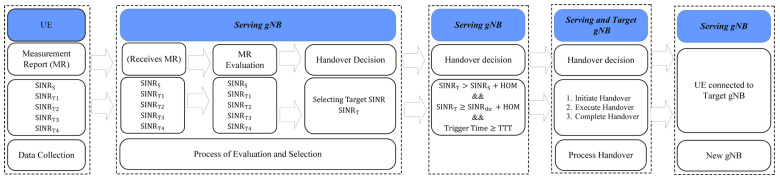
Model structure of the HOD algorithm based on SINR functions for 5G/6G networks.

In this paper, an SINR-based HO decision-making system model is presented as an important part of designing and improving wireless communication networks. The proposed model is designed to facilitate better connection quality, network intelligence, and user mobility support, all of which are key to fulfilling the potential of 5G and future 6G networks. The model uses a dual-condition handover algorithm to guarantee seamless connectivity and minimize the ping-pong effect between cells, as illustrated in Figure 5. The HO process starts with measuring the SINR and reporting this to the base station (gNB) that controls the HO process by the user equipment (UE). The data is then filtered and processed in the measurement evaluation block to stabilize and enhance accuracy. A two-step target cell selection process is adopted: first, checking the availability of resources in the target cells; second, comparing the SINR with RSRP. The target cell with the best SINR and sufficient resources will be selected.

The selected target cell for HO will always have sufficient resources, which will boost user throughput. The proposed method for the HOD process is based on two decision algorithms, each chosen depending on available resources. In the first decision process, the HOD is based on the HOM, which is initiated, and the SINR of the serving and target cells, assuming that the target cell has more resources than the serving cell.

Additionally, the target cell’s SINR must exceed the specified thresholds. The second decision process states that if the target cell has more resources available than the serving cell, the HOD is based exclusively on the HOM, the target cell, and the threshold SINR levels. When both conditions are met, an HO is deemed necessary, triggering the HO execution module, which orchestrates signaling and procedures to seamlessly transfer the UE’s connection to the HO (target cell), ultimately connecting the UE to the System.

The decision criteria are expressed as follows:

(3) $${SINR}_{T}\geq {SINR}_{S}+HOM\;\&\&\;{SINR}_{T}\geq {SINR}_{thr}+HOM$$

where ${SINR}_{S}$ and ${SINR}_{T}$ represent the SINR of the serving and target BSs, and ${SINR}_{thr}$ represents the threshold of SINR. HOM indicates the fixed HOM (dB) value by selecting a random value available in the serving cell.

Accordingly, the SINR-based handover decision function is defined as

(4) $$HOD\left(t\right)=\{\begin{array}{cc} 1, & \;\;if\;{SINR}_{T}>{SINR}_{S}+HOM\;and\;{SINR}_{T}\geq {SINR}_{thr}+HOM\\ 0, & \;\;\;\;\;\;\;otherwise \end{array}$$

where HOD(t)=1 indicates that the HO is executed, and HOD(t)=0 indicates that the UE remains connected to the serving cell.

The proposed SINR-based dual-condition HO improves mobility awareness in 5G/6G networks, simultaneously improving SINR and verifying minimum SINR quality before triggering the HO process. The first condition in Equation (3), ${SINR}_{T}>{SINR}_{S}+HOM$, ensures the target cell provides higher signal quality compared with the serving cell. This condition avoids unnecessary HOs caused by small SINR fluctuations that can reduce the ping-pong effect. In contrast, the second condition in Equation (3), ${SINR}_{T}\geq {SINR}_{thr}+HOM$, ensures that the target cell satisfies the minimum requirement for stable communication. Further, the second condition prevents HO execution that can cause a weak or highly interfered cell to result in radio link failure. Additionally, the proposed algorithm achieves greater stability and a more reliable HO decision than traditional HOs. The dual condition improves mobility performance, minimizes HO failures and unnecessary HOs, and enhances service quality. In 5G/6G ultra-dense networks, the proposed SINR-based HO provides better mobility awareness and adaptability. Furthermore, this approach helps reduce HO ping-pong and enhances system throughput and seamless connectivity at high mobile speeds.

## 4. System Model

This section examines the network deployment scenario in ultra-dense cellular networks and system settings, the mobility and traffic models, and the measurement report for 6G networks. We considered the urban environment of 5G/6G networks to evaluate HO decisions using a mobility-awareness model.

### 4.1. Network Deployment Scenario

**Figure 6:**
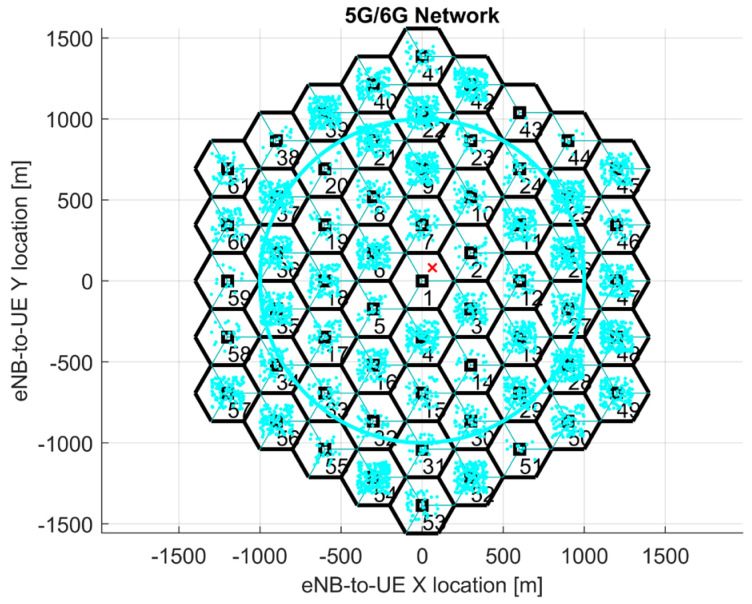
Simulation environment for 5G/6G cellular networks.

The design of Figure 6 consists of 61 small cells. Each small cell contains three sectors, which consist of 183 small cells known as base stations. Blue dots indicate fixed users and are randomly distributed throughout the communication network. The cross point shown in cell number one is considered a user. These mobile user measurements are fixed according to their position. The mobility scenario in this study involves users moving in fixed directions from 0 along the axes (+X). Based on our understanding, employing a unidirectional mobility model is superior to using a random one. This is because it allows users to traverse multiple base stations more quickly, increasing the number of handovers during the simulation. In our symmetrical system environment, the value of 0 is irrelevant. Furthermore, this model more accurately replicates actual network scenarios. However, in the random mobility model, users are likely to remain in the same area based on probability. Consequently, the HOP is still low. In the random mobility model, users have the ability to move from one base station to another and traverse multiple cells. These movements are still probabilistic. The significance of this study for network engineers and wireless communication researchers is that it provides insights into the effectiveness of the various mobility models.

The trajectory of the test subjects studied is marked by the black arrow in Figure 6. In this study, five UEs were tested at different starting positions (x, y) at various speeds in the cellular network. The movement steps were adjusted for each simulation cycle based on the speed level. The faster the mobile speeds, the more steps the mobile will take, which will impact the HCPs. Therefore, minimizing the HCPs is necessary. In addition, six mobility scenarios were examined at speeds of 40, 60, 80, 100, 120, and 140 km/h.

### 4.2. Mobility Model

The mobile user is randomly located in the network environment at a small cell. Small BSs are allocated to various mobile users, and the small cells are represented by blue and black dots, with the blue dots representing users. Mobile users, however, move in one direction, and each user will follow different parallel lines than the other chosen users. The active users (five users) are moving in parallel directions to ensure that they are aligned. The directional mobility model of user mobility was chosen since it makes it easier to analyze various HO scenarios. Moreover, the performance of the users is also evaluated at a single point in the simulation cycle, and the system is reliable; the interval of 40 ms corresponds to the distance of the mobile user’s movement.

### 4.3. Channel Model

5G combines multiple cell tiers operating simultaneously to meet the dense-capacity requirements of urban coverage. However, developing an accurate model of radio channels in network deployments remains a critical challenge for system design, interference management, and performance evaluation. We consider the propagation model for urban areas suitable for scenarios in urban regions beyond the high-rise core, where buildings have approximately uniform heights. The path loss model for a cellular network with carrier frequency f in an urban area for a connection between a gNB and a mobile user (UE) is expressed in decibels (dB) [7,38]. The mathematical formula for the path loss is given as follows:

(5) $$P_{L}=\;40\;\times \;(1\;-\;4\;(10^{-3})d)\log_{10}\left(R\right)-\;18\;\times \;\log_{10}\left(d\right)+\;21\;\times \;\log_{10}\left(f\right)+80\;\mathrm{dB}$$

where *R* is the base station (gNB) user distance in kilometers (Km), f is the carrier frequency (f = 28 GHz and f=120 GHz) in MHz, and d is the antenna height of the BS in meters.

### 4.4. Traffic Models

In wireless networks, traffic models for UEs are designed to predict user mobility, network performance, call patterns, duration, HO, and resource allocation across macro- and small cells. However, they enable accurate performance, handover optimization, and effective capacity planning. In this context, we propose two systems to evaluate network performance based on user movement, including HOP, HOPP, RLF, and data rates. These metrics depend on different mobile speeds and the distance between gNBs in urban areas. Network performance is determined by the frequency with which a user crosses a cell boundary. Additionally, network deployment scenarios aim to analyze SINR using the algorithmic functions in Equation (3) to ensure reliable connectivity and higher data rates, which are increasingly important in 5G/6G networks.

### 4.5. Network and Systems Settings

The structure of Figure 6 presents a 5G/6G network model covering a 9 km^2^ study area in an ultra-dense environment. The simulation study implemented an HOM with mobility awareness for the network model environment. The network systems are modeled based on radio-frequency scenarios in 3GPP Release 19. However, the global system is simulated using the highly reliable MATLAB 2023a software, ensuring accuracy and addressing various HCPs. Although 183 small cells are deployed using 46 dBm transmission power, the input measurement report (RSRP, SINR, and UE speed) is evaluated within the simulation area to provide the output results for HOM and TTT. The selected users (five UEs) are displaced in random directions across various mobile speed scenarios ranging from 40 km/h to 140 km/h. Furthermore, the HCPs (HOM and TTT) are selected based on this study to balance HO responsiveness and reduce HO ping-pong. Moreover, 28 GHz and 120 GHz operating frequencies are widely used for small BSs for network 1 and network 2, respectively. Table 2 summarizes the main parameters used in the simulations. These parameters are based on 3GPP Release 19 [39].

### 4.6. Simulation Model

**Figure 7:**
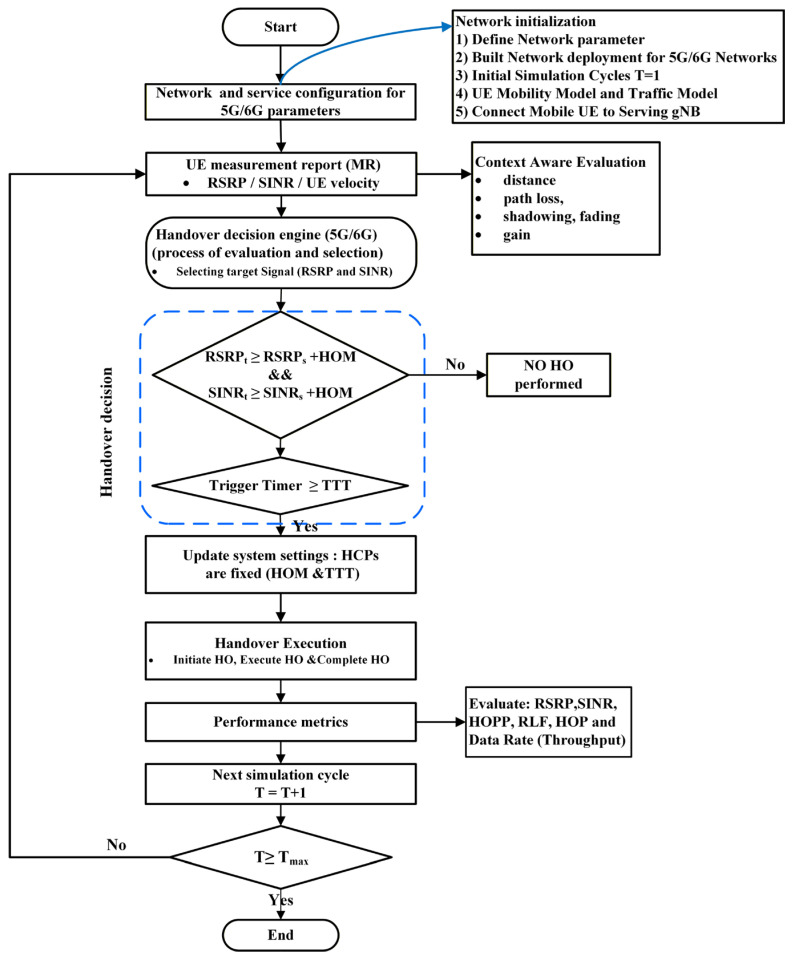
Simulation flow chart for handover decisions in a cellular network.

Figure 7 represents the simulation flow chart for 5G/6G network. This study begins by identifying the network parameter presented in Algorithm A1 (see Appendix A). The process starts with the network coverage area zone, followed by the network mobility model. Additionally, each user’s position and direction are updated every 40 milliseconds (i.e., a simulation cycle). Furthermore, the Euclidean distance from each simulation cycle’s BSs to each user’s BSs is evaluated. Additionally, shadowing is added to the obtained distances to estimate the path loss along the propagation path. The HO decision process is described in Figure 7 [24].

## 5. KPI Formulation and Analysis

The literature review discusses the HO performance of the system network, as established by the KPIs. Our simulation outputs in this paper are carried out under the following conditions for RSRP [14], SINR, HOP, HOPP, RLF, and data rate [24,40].

### 5.1. Received Signal Reference Power (RSRP)

RSRP is a measurement of the received power in wireless access networks. The average signal strength, also known as the average RSRP, indicates the power level or strength with reference to a given signal. In LTE networks, RSRP is calculated by averaging the total power of the cell-specific reference signal resources carrying the cell-specific reference signals. The 5G and 6G network synchronization signals are derived from the secondary synchronization signals:

(6) $$RSRP\;\left(dBm\right)=PTx\left(dBm\right)+G\left(dB\right)+G_{UE\left(dB\right)}+P_{L}+fading$$

where PTx is the transmission power of the MC, G(dB)  is the antenna gain direction applied, $G_{UE\left(dB\right)}$ is the user gain, $P_{L}$ is the path loss for the MCs, and fading denotes fast fading.

### 5.2. Signal to Interference Plus Noise Ratio (SINR)

The SINR is a model that compares the signal power to the total interference-plus-noise power. The minimum SINR is the ratio of the required signal power to the interference-plus-noise power needed to achieve successful packet reception in wireless communication networks:

(7) $$SINR\;\left(dB\right)=10*\log_{10}\left({SINR}_{ratio}\right)$$

(8) $$\mathrm{where}\;{SINR}_{ratio}=\sum \frac{{RSRP}_{s}}{I_{rx}+N_{0}}$$

where $I_{rx}$ is the interference power, and *N*_0_ is the thermal density noise.

### 5.3. HO Probability (HOP)

HOP: HOP is used to denote the likelihood of HO during the transition of the user equipment between cells. It determines the ratio of HO events taking place. The HOPP is one of the factors that increase HOP. It helps reduce HOP, thereby lowering the signaling overloads of the system, in addition to conserving network resources. The HOP average will be determined at each UE in the system during each simulation cycle:

(9) $$\bar{HOP}=\frac{\sum_{i=1}^{N_{UE}}\;P_{i}(HO)}{N_{UE}}$$

where $P_{i}(HO)$ is the HO probability ratio, and $N_{UE}$ is the total number of UE in the system.

### 5.4. HO Ping Pong (HOPP)

The HOPP is the repetitive HO that exists between two neighboring cells. The high frequency of the UE between the two successive cells creates a ping-pong effect as a result of a large difference in signals. The HOPP is calculated as a ratio of the total number of HOOPs:

(10) $$HOPP=\frac{N_{HOPP}}{N_{HO}}$$

where $N_{HOPP}$ is the HOPP ratio, and $N_{HO}$ is the total number of HOs (HO failures and successes).

### 5.5. Radio Link Failure

A radio link failure is a situation that occurs when the reverse HO connection to the source cell fails. In such a case, the failure denotes that, even when there are good radio conditions to decode the reports of the measurements transmitted by the UE to the source gNB and the following signals are transmitted to the target cell to carry out the HO, the UEs do not decode the received HO order by the source gNB. It should be noted, though that when an RLF is detected during an HO, the UEs provide a recovery mechanism that validates the system’s reliability. The RLF is computed using the average probability of RLRP of all mobile users:

(11) $$\bar{RLFP}=\frac{\sum_{i=1}^{N_{UE}}RLF(i)}{N_{UE}},$$

where i denotes the number of users, and $N_{UE}$ denotes the total number of all users measured.

### 5.6. Data Rate (Throughput)

The data rate refers to the amount of data transmitted from one location to another within a designated time frame. It is a variable contingent on the availability and effective use of network resources. In wireless networks, increased throughput is achieved by optimizing resource utilization. In congested cells, variations in data rates may arise from resource scarcity, adversely impacting users’ QoE:

(12) $$R_{bits}^{UE}=\;\frac{Total\;bits\;transmitted\;to\;UE\;in\;one\;subframe}{subframe\;duration\;(T_{SF})}$$

The total bits per frame contains the number of resource blocks (RBs) assigned to the user.

$$N_{RE}^{RB}=N_{SCs}^{RB}*N_{symbols}^{NC}$$

Also, $N_{data}^{RB}=\;N_{SCs}^{RB}*(N_{symbols}^{SC}-N_{RE}^{RB})$

By applying the modulation symbol carrier and code rate (CR), we obtain

$$Useful\;bite\;per\;RB=N_{SCs}^{RB}*\left(N_{symbols}^{SC}-N_{RE}^{RB}\right)*m_{bits}^{symbol}*CR$$

The total bits per subframe is

(13) $$R_{bits}^{UE}=\frac{{{N_{CCs}^{UE}*N}_{RBs}^{UE}*N}_{SCs}^{RB}*\left(N_{symbs}^{SC}-N_{RE}^{RB}\right)*m_{bits}^{symb}}{T_{SF}}CR$$

where the equation is composed of the following: $N_{CCs}^{UE}$: carrier components; $N_{RBs}^{UE}$: RBs per user; $N_{SCs}^{RB}$: subcarriers per RB; $N_{symbs}^{SC}$: OFDM symbols per subframe; $N_{RE}^{RB}$: reference symbols per subcarrier; $m_{bits}^{symb}$: modulation bits; CR: coding rate; $T_{SF}$: subframe duration.

## 6. Results and Discussion

The results of the simulation study are presented in this section, using MATLAB 2023a simulations to investigate and validate the implementation of the proposed method. The network system was examined under six mobile-speed scenarios to evaluate each user’s performance during the simulation cycle.

### 6.1. Evaluation of Measurements for RSRP and SINR

In this section, the simulation study evaluates the RSRP and SINR measurements to support the proposed handover decision algorithms.

#### 6.1.1. Performance Based on RSRP

This subsection presents and analyzes the RSRP results obtained from our study. The average CDF and RSRP for all evaluated users are shown in our study. These findings demonstrate the effectiveness of various automatic self-optimization methods across different mobility speed scenarios. The results demonstrate that no single algorithm can achieve optimal RSRP performance across all mobile-speed conditions.

The UE periodically measures the RSRP from all available BSs and sends the measurement reports to the serving BS. The HO will only occur if the necessary conditions are fulfilled; otherwise, the UE will stay with the serving BS and keep monitoring the signal. Optimization of the HCPs is done based on the received signal strength for the improvement of the HO’s performance. At higher RSRP, lower HCPs values are set to maintain the connection, and at lower RSRP settings, proper HCPs settings can help make HOs smoother. The values for TTT and HOM are fixed in this study for the RSRP-based system, whereas the RSRP-based method is applied to all network parameters.

**Figure 8:**
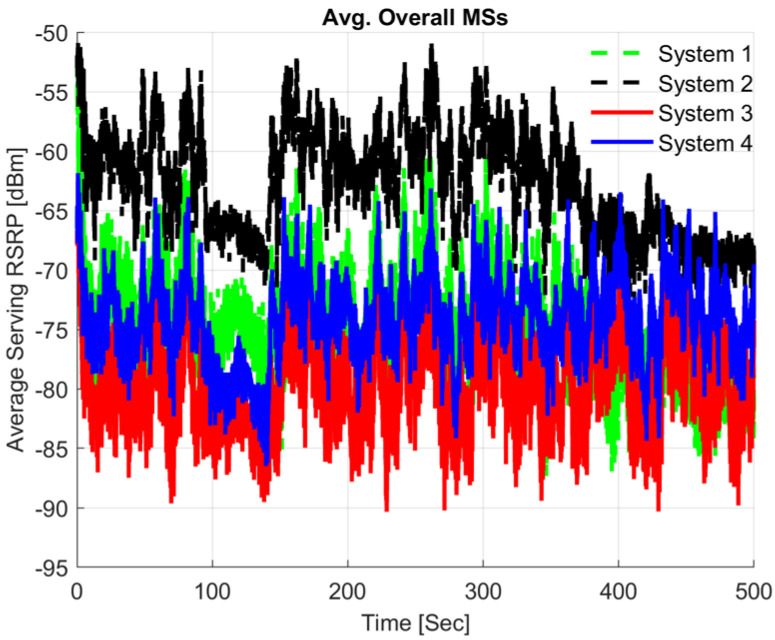
Average RSRP overall mobile speed scenarios over simulation cycles.

Figure 8 illustrates the average RSRP overall mobile speed over 500 s of simulation cycles. The systems presented a comparison of the HO decision algorithm for all systems based on RSRP and SINR to assess the average RSRP overall mobile speeds (40–140 kmph) for all users. The results indicate that Systems 2 and 4 use the HO decision approach, which provides the best RSRP value across all mobile speeds. At low speeds (40–100 kmph), Systems 2 and 4 provide more stable RSRP values for the UEs in the ranges of −58 to −70 dBm and −60 to −75 dBm, respectively. However, in Systems 1 and 3, the overall average RSRP decreased as the mobile speed and simulation cycle increased. Thus, Systems 1 and 3 decreased due to the UE’s movement across the boundary cell. Furthermore, Systems 2 and 4 maintain the highest RSRP, and the UE remains connected to the serving cell.

**Figure 9:**
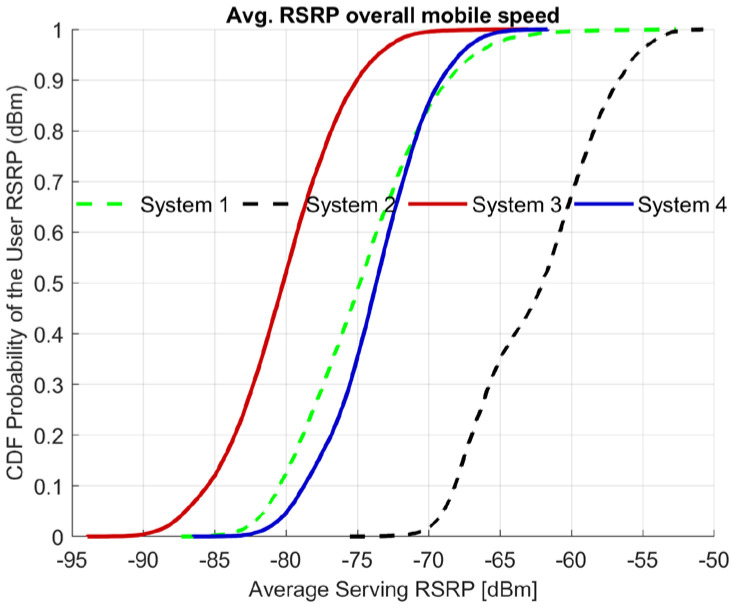
Average CDF of RSRP overall mobile speed scenarios.

Figure 9 displays the CDF of RSRP across different mobile speeds for all users. The results demonstrate the reliability of the HO decision algorithm under different mobility scenarios. At all speed levels, Systems 2 and 4 attain higher RSRP values than Systems 1 and 3. Both Systems 2 and 4 offer better signal quality in their coverage area at slower speeds (40–80 kmph). At higher speeds (100–140 kmph), the performance of Systems 1 and 3 decreases, with more signal changes, resulting in a degradation of the user experience. The serving cell will continue to provide a stronger signal to System 2 for a longer period of time, and System 4 is stable. Overall, System 2 will have better coverage and will be stronger than System 4 at various speeds, resulting in a higher RSRP.

**Figure 10:**
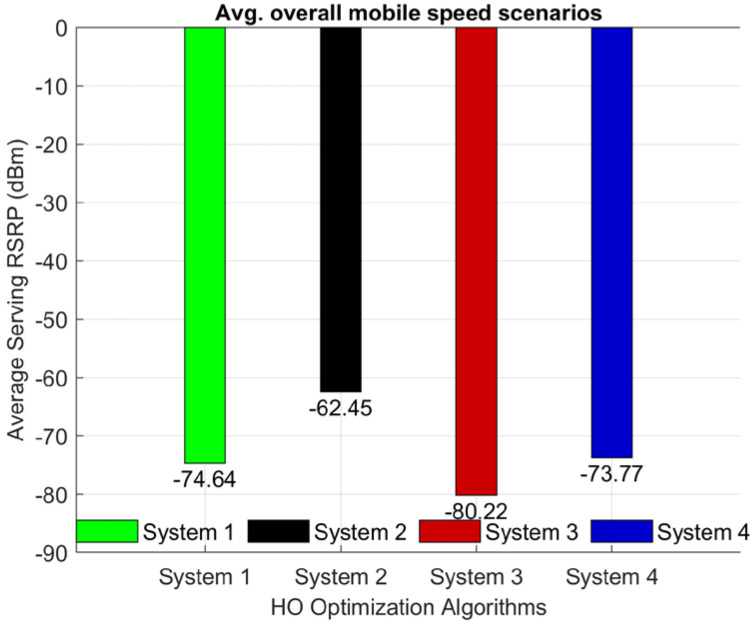
Average RSRP overall simulation time under various mobile speed scenarios.

Figure 10 highlights the average RSRP overall mobile speeds and simulation cycles. The results show that at lower speeds (40–80 kmph), System 1 and System 4 maintain minimum RSRP levels above −60 dBm and −73 dBm, respectively. At higher speeds, the effect on RSRP is that the speed increases from 100 to 140 kmph, and the RSRP level gradually decreases. However, the result shows that the system maintains signal quality from serving cells during mobility based on RSRP. Overall, Systems 2 and 4 perform better than Systems 1 and 3, with average RSRP values of −62.45 dBm and −73.77 dBm, respectively, compared to −74.63 dBm and −80.22 dBm in Systems 1 and 3, respectively.

#### 6.1.2. Performance Based on SINR

**Figure 11:**
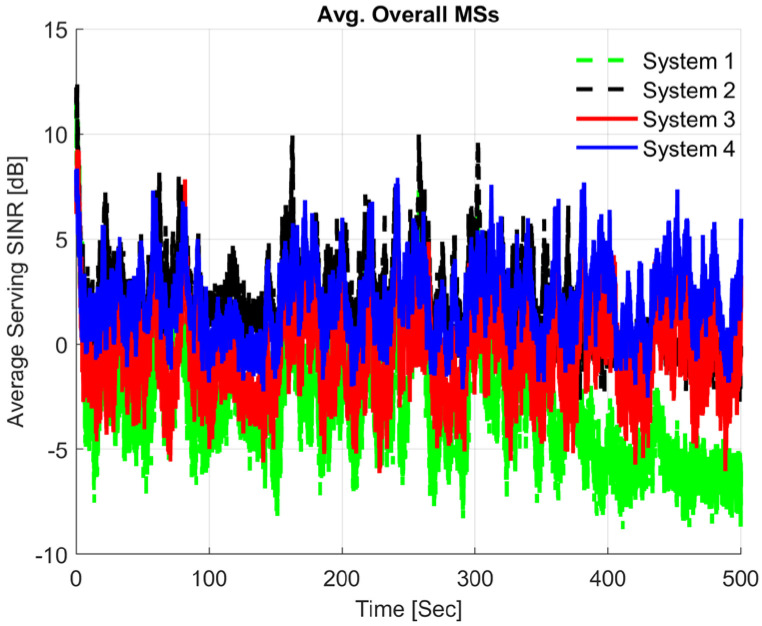
Average SINR overall mobile speed scenarios over simulation cycles.

In this subsection, the SINR results are illustrated and discussed. Figure 11 demonstrates the average SINR overall mobile-speed scenarios over the simulation cycle. At low speeds (40–80 kmph), Systems 2 and 4 provide a stable signal that allows user movement with a permanent signal, while Systems 1 and 3 relatively maintained performance at low SINR values. As speeds increase, System 1 provides a low signal due to increased noise and interference, while System 3 decreases. Additionally, System 4 provides a higher SINR level that enhances interference management and stability for user mobility, with stronger, more frequent SINR than Systems 1 and 3. However, at high speeds, all systems in both networks experience signal degradation due to severe fading.

**Figure 12:**
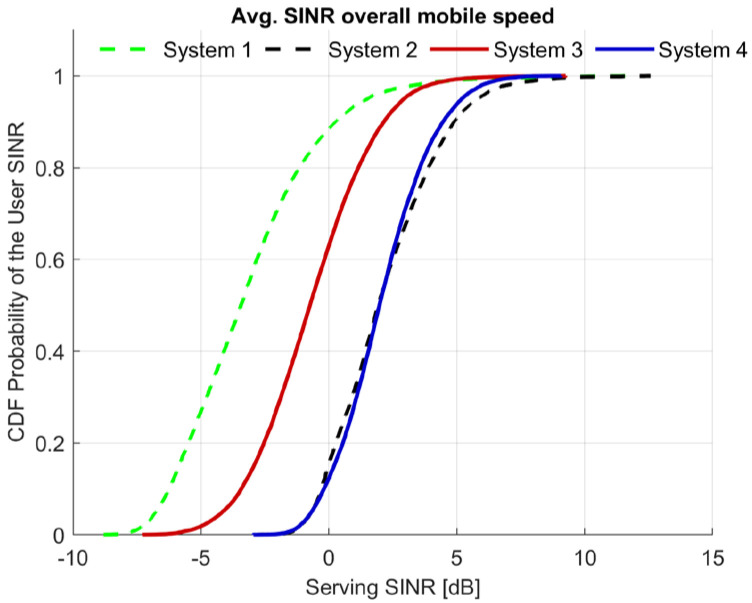
Average CDF overall mobile speed scenarios and simulation cycles.

Figure 12 displays the average CDF of SINR for all systems across two networks and all measured users and mobile-speed scenarios (40–140 kmph). The results visualize system performance across various mobile speed scenarios. The results reveal that Systems 2 and 4, based on the SINR algorithm, achieved the highest SINR levels, with averages across all mobile speed scenarios. All mobile speeds from System 2 are significantly higher than those from System 4, and its SINR is good compared to Systems 1 and 3. However, the mean enhancement value is low for Systems 1 and 3 due to their worst performance under mobile-speed scenarios. Furthermore, Systems 2 and 4 across all mobile speeds maintain an advantage and exhibit robustness, with high SINR performance.

**Figure 13:**
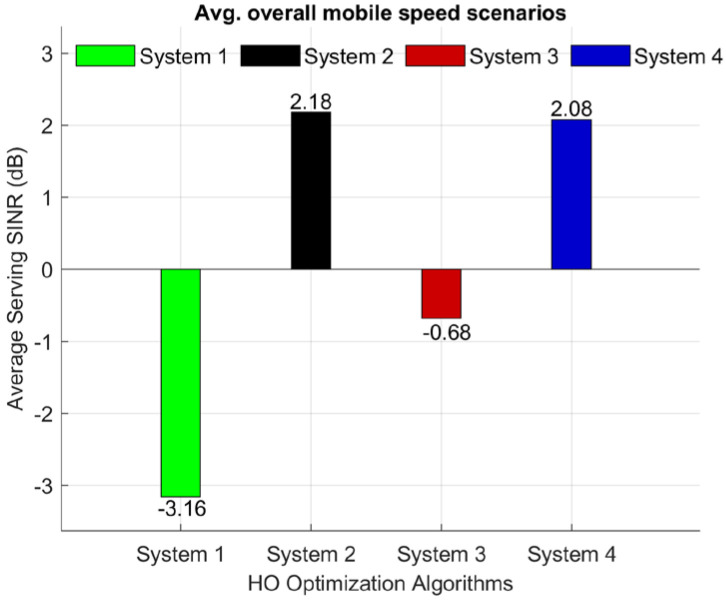
Average SINR overall simulation time under mobile speed scenarios.

Figure 13 represents the average serving SINR over all mobile users and simulation cycles for all mobile speed scenarios. The result indicates that the minimum SINR across all speeds exceeds the SINR thresholds for all systems. When the mobile user is moving at mobile speeds, Systems 2 and 4 provide better signal performance than Systems 1 and 3. This case shows that it outperforms Systems 2 and 4 across overall mobile speeds by 2.18 dB and 2.08 dB compared with Systems 1 and 3, which are −3.16 dB and 0.68 dB.

Therefore, the results indicate that Systems 2 and 4 are relatively sensitive to user mobility and have a more consistent SINR than Systems 1 and 3.

### 6.2. Evaluation of Handover Performance

The proposed HO decision algorithm is evaluated by comparing two networks (5G and 6G), each composed of two systems based on RSRP and SINR, respectively. The HO performance is assessed via simulation across deployment network scenarios in a 3 km × 3 km area. The user mobility speed ranges from 40 to 140 kmph over the simulation cycles, and the user measurement period is 40 ms. We simulated two HO decision algorithms in the A3 event with a fixed HCP of HOM = 3 dB and TTT = 160 ms for the two networks. The proposed HO decision algorithm outperforms System 2 and System 4 in terms of HOOP, RLF, and data rate, and it also shows improved performance in the measurement reports for RSRP and SINR, as shown in Section 6.1.1 and Section 6.1.2, respectively. Table 3 presents the overall average HO performance of the evaluation system settings for all users (five users) across mobile speed scenarios from 40 to 140 kmph across the simulation cycle. From Table 3, Systems 2 and 4 in 5G and 6G achieve the best performance, demonstrating the performance of the proposed algorithms.

#### 6.2.1. Performance Based on HOP

**Figure 14:**
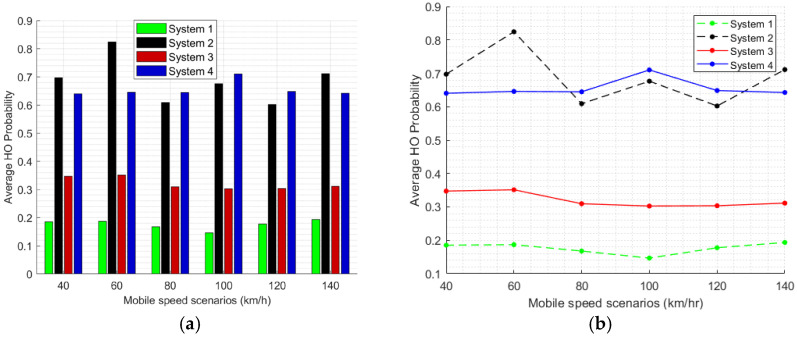
(**a**) Average HOP probability versus mobile speed scenarios for all users. (**b**) Average HOP probability versus mobile speed scenarios for all users.

Figure 14a,b illustrate the average HOP probability for all users in Networks 1 and 2 (RSRP and SINR) under mobile speed scenarios. However, the system establishes a low number of HO. It becomes more robust while unnecessary HOs are reduced. Additionally, across mobile speed scenarios, Systems 1 and 3 consistently exhibit a lower HOP than Systems 2 and 4, resulting in fewer HO events and higher mobility at low mobile speeds (40–100 kmph). As the mobile speeds increase beyond 100 kmph, the HOP increases for Systems 2 and 4 and remains consistently low for Systems 1 and 3. However, the reduction is more proportional for Systems 2 and 4. Furthermore, Systems 1 (5G network at 28 GHz) and 3 (6G network at 120 GHz) clearly exhibit low HOP across mobile speeds, with minor fluctuations due to increased interference. Therefore, Systems 2 and 4 provide higher HOP efficiency and robustness under mobility conditions than Systems 1 and 3.

**Figure 15:**
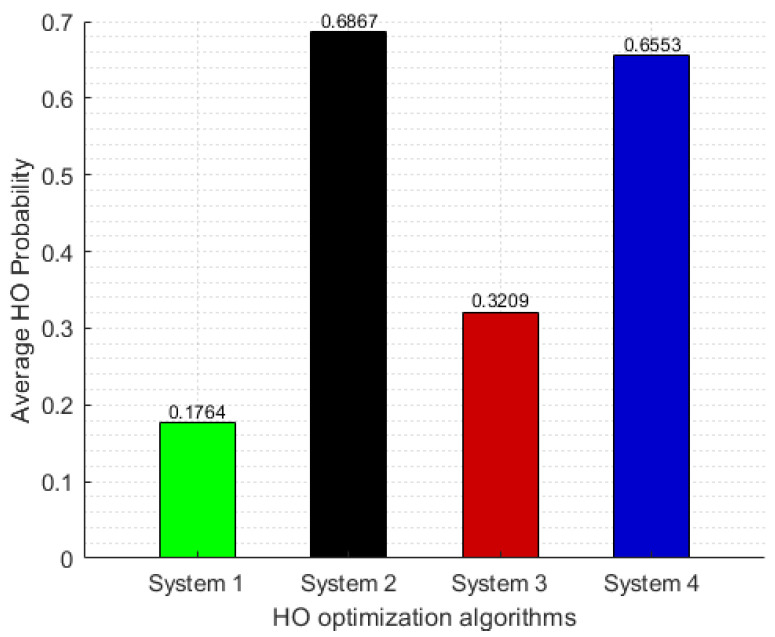
Average HO probability versus HO optimization systems.

In Figure 15, the results show the average HOP for the two HO algorithms for all systems under mobile-speed scenarios. *The figure* illustrates a reduction in the HOP rate, especially when comparing four systems for a network environment. However, Systems 2 and 4 have the highest average HOP over all mobile-speed scenarios. Additionally, the average reduction gains for the HO algorithm for Systems 1 and 3 are around 17.64% and 32.09%, and for Systems 2 and 4, they are 68.67% and 65.53%, respectively.

#### 6.2.2. Performance Based on HOPP

The HO ping-pong probability is an important key performance indicator that may result from the initial HCP setting. The HPPP is triggered by establishing minimal thresholds by assigning the values of TTT and HOM.

**Figure 16:**
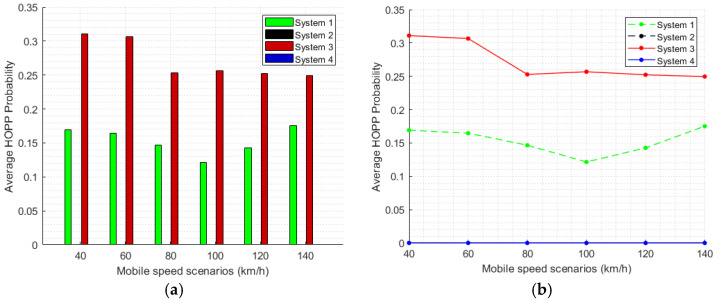
(**a**) Average HOPP probability versus mobile speed scenarios for all users. (**b**) Average HOPP probability versus mobile speed scenarios for all users.

Figure 16a,b represent the average HOPP probability for four systems under different mobile speed scenarios ranging from 40 km/h to 140 km/h for all users. However, two handover decision metrics are compared, RSRP-based and SINR-based metrics, with each represented for two networks (5G and 6G). The results indicate that the HOPP probabilities for Systems 1 and 3 are higher, particularly at low speeds up to 100 km/h, where users remain longer in the serving cell. With higher mobile speeds, the probability of an HOPP decreases slowly for all systems. All systems use fixed HCPs, such as trigger TTT and HOM, which impact radio link failure (RLF) performance. However, Systems 2 and 4 have lower HOPP probabilities than Systems 1 and 3. Therefore, HOPP should be minimized to preserve network resources.

**Figure 17:**
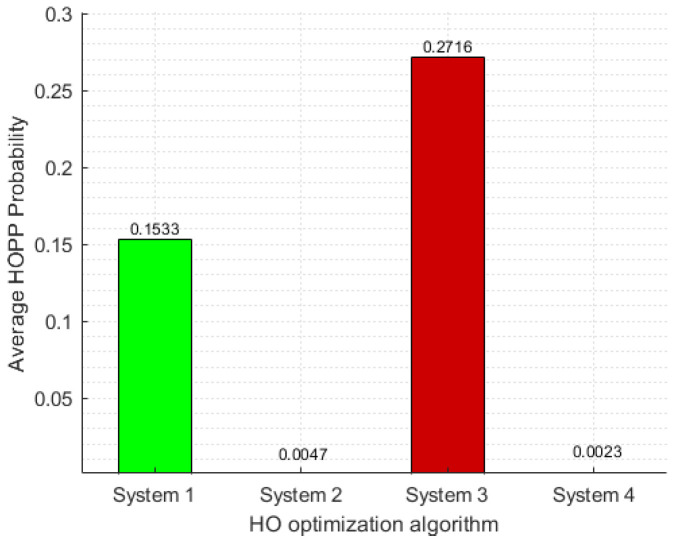
Average HOPP probability versus HO optimization systems over mobile speeds scenarios.

Figure 17 represents the HOPP probability compared with the HO algorithm across a wide range of simulation times and mobile-speed scenarios. The proposed algorithm for Systems 2 and 4 demonstrates robustness, with user performance gains of approximately 99.97% and99.99% across diverse mobile speed scenarios and simulation times for HOPP. The results further indicate that Systems 1 and 3, based on the HO algorithm, respond more to mobile speed scenarios and to optimizations updated over time.

Additionally, high HOPP activity creates unstable connections and indeterminate resource usage. Moreover, Systems 4 and 2 have low HOPP and remain stable in performance. Table 3 presents the performance of the systems when applying fixed HCPs. It can be observed that Systems 2 and 4 show the best performance compared to Systems 1 and 3. This case significantly reduces ping-pong events over time; maintains consistent handover stability across varying network settings; and also enhances network efficiency, minimizes signaling overhead, and provides better user experience.

#### 6.2.3. Performance of RLF

RLF represents the potential for a disruption in the radio link between the user and the service network during an active call, an occurrence that depends on multiple triggering events. This is similar to HOPP, with the proposition of the HCP setting being defined or estimated. Additionally, the HCP has been set to 160 ms for TTT and 3 dB for HOM in both systems. Unlike HOPP events, RLFs arise from prioritizing TTT and HOM, particularly for users engaged in high-speed events or near the cell boundary. However, RLF should be minimized to improve system performance and optimize network resources.

**Figure 18:**
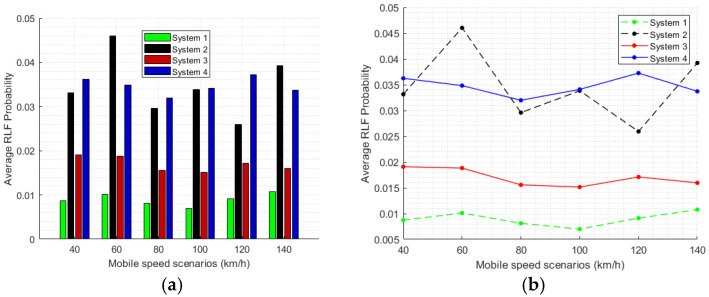
(**a**) Average RLF probability versus mobile speed scenarios for all users. (**b**) Average RLF probability versus mobile speed scenarios for all users.

Figure 18a,b illustrate the average RLF probability for users under six different mobile speed scenarios over all simulation cycles. With this result, the evaluations are presented in Table 3 as the average RLF probabilities across all users and mobile speed scenarios for both systems. The simulation results in Table 3 show that Systems 2 and 4 have higher RLF probabilities than Systems 1 and 3 at all mobile speeds, indicating lower RLF reliability with increasing mobility. As shown in Table 3, the HOPP value, which is inversely proportional to the RLF probability, further indicates that System 1 reacts to the simulation time.

**Figure 19:**
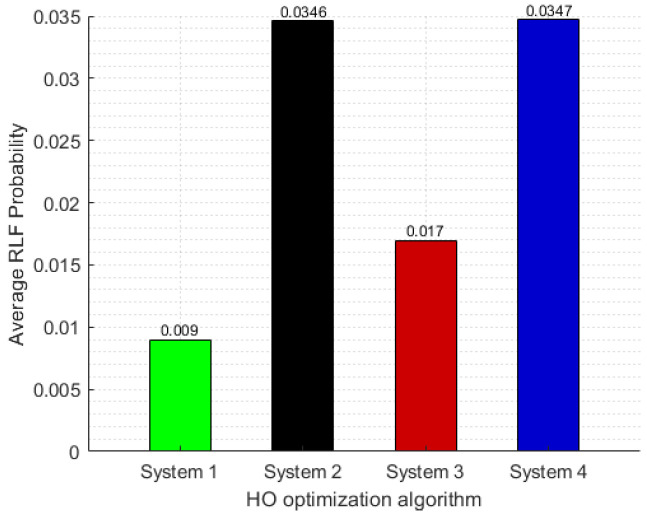
Average RLF probability versus HO optimization systems.

Figure 19 presents the average RLF probability for all users of the HO optimization algorithm over mobile speed scenarios and simulation cycles. Although the average reduction gain performance of the RLF probability, evaluated by the RLF probability, for System 1 and System 3 achieved 74.32% and 51.01%, respectively. However, the proposed algorithm shows a reduction in the performance gain of the RLF compared to the HOPP, with 99.97% for System 2 and 99.99% for System 4. The results further show that the RLFs vary over time across all mobile speed scenarios. The results also show that the systems for all algorithms performed consistently over time.

#### 6.2.4. Performance Based on Data Rate

Throughput is also referred to as the data rate because it is the rate at which data is transferred between a cell tower or base station and a mobile device, e.g., a smartphone. It is an important performance measure for a wireless communication system and is usually measured in bits per second (bps), kilobits per second (Kbps), or megabits per second (Mbps). The rate of cellular communication networks can be affected by several factors, including network congestion, signal strength, interference, network technology, bandwidth allocation, device capabilities, and QoS. Nonetheless, the data rate is a critical factor for mobile users who need fast, high-quality data connections in their network operations.

**Figure 20:**
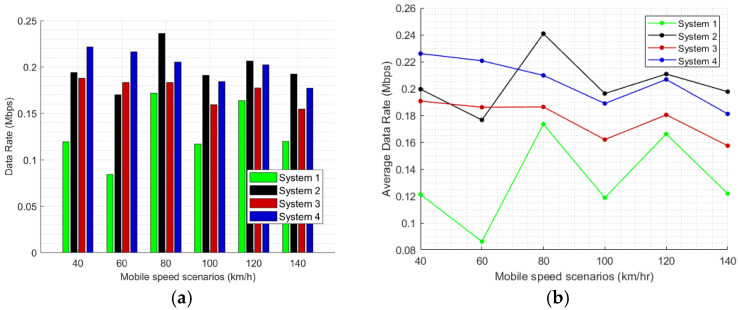
(**a**) Average data rate for user mobile versus mobile speed scenarios. (**b**) Average data rate for user mobile versus mobile speed scenarios.

Figure 20a,b display the average data rate of different mobile scenarios for all measured users and the data rate for all simulation cycles. The results show that, compared to HOD techniques, the proposed algorithm improves the average throughput across all speed scenarios. The average data rate in Table 3 indicates that Systems 2 and 4 achieve the highest data rate compared to Systems 1 and 3. The results indicate that System 2 provides a significant increase in data rate across all mobile speed scenarios, while System 4 achieves the highest performance with a strong SINR value. This is a noteworthy accomplishment: Systems 2 and 4 improve the UE data rate by considering resource availability and SINR levels in target cell selection and HODs.

**Figure 21:**
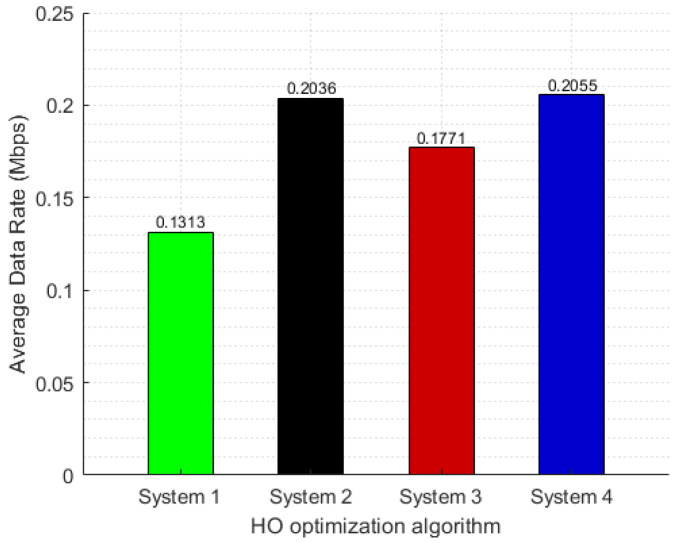
Average data rate probability versus HO optimization systems.

Figure 21 displays the average data rate for all users and various mobile speed scenarios over simulation cycles. The results compare all systems with two networks as the frequency varies. Systems 2 and 4, which use the SINR-based algorithm, improve the average data rate across all speed scenarios. The overall average throughput performance for Systems 2 and 4 improves by 55.11% and 16.004%, as shown in Table 5. The result shows that Systems 2 and 4 improve users’ performance by having the edge cell supply more capacity frames, reducing RSRP while maintaining a high threshold.

Table 4 presents the system performance evaluation based on KPIs for all systems, considering five UEs and various mobile-speed scenarios over 20,000 simulation cycles. The results demonstrate distinct differences between the systems. System 2 provides a higher data rate than System 1, with data rate values of 0.20361 and 0.13127, respectively. Also, System 4 achieves the maximum data rate of 0.2055, exceeding System 3 at 0.1771. Furthermore, the HOPP ratio is lower for Systems 2 and 4, suggesting higher handover stability. All KPI improvements are also shown in Table 5. The analysis shows the performance gains in Systems 2 and 4, especially in RSRP, SINR, and data rate. System 2 also significantly decreases the number of HOP and increases mobility by decreasing both the number of HOP and RLF in System 1.

## 7. Conclusions

This paper compared pairs of systems using an HOD algorithm to evaluate network performance in 5G and 6G wireless networks. This study focuses on the required RSRP and SINR conditions for the target cell and all mobile users, which improves network performance (throughput and large bandwidth) during the handover process. This paper came up with a two-system target-cell approach that uses RSRP and SINR. The 5G/6G mmWave technologies that meet the RSRP and SINR requirements are selected when choosing target cells. The mobility management plan in the HOD is beneficial to the performance of the system. This is followed by prioritizing the association of mmWave cells with users across various HO processes depending on the serving cell’s status and cell type. Two system scenarios with fixed HOM and TTT values were analyzed to examine the impact of HCPs on the performance of the proposed HOD algorithm. The results show that these parameters strongly influence the system efficiency of 5G-A and 6G networks in ultra-dense environments. The HOD performance criteria for the KPIs, including serving-cell RSRP and SINR, HOP, HOPP, RLF, and data rate (throughput), have been discussed and analyzed. Additionally, the proposed algorithm was implemented across different mobile-speed scenarios in 5G/6G networks. The simulation results indicated that the proposed method clearly shows significant performance improvement across systems. The simulation results for Systems 2 and 4 showed improvements in signal quality (RSRP and SINR) and data rate, respectively, while Systems 1 and 3 showed reduced HOP and RLF. Furthermore, Systems 2 and 4 outperform Systems 1 and 3. An intelligent handover decision algorithm based on ML for future mobile networks in smart cities will be our target in future research, particularly for heterogeneous and IoT networks in smart cities.

## Figures and Tables

**Table 1:** Summary of related studies.

Ref.	Problems	Network	HOD Algorithm	Optimization Parameters	KPI’s
[30]	Limitations of optimization HO decisions	5G and ultra-dense HetNets	DL-based HO in the RSRP-based	RSRP	➢ Energy consumption
[31]	Inefficient vertical HOD in HetNet, which provides high HO rates, HOPP, delay, and costs.	LTE	MADM uses TOPSIS in the RSS-based	TTT	➢ HOR ➢ HO delay ➢ HO cost ➢ Energy ➢ HOF
[32]	Low HO performance in HetNets leads to high call blocking and unnecessary HO, making optimal HO difficult due to the diversity of networks.	Wi-Fi and LTE	Adaptive HOM/TTT-based RSS	TTT	➢ HO call blocking ➢ Data rate ➢ Energy ➢ HO failure ➢ HO delay
[33]	High-quality mobile connectivity for m-health applications for user mobility in 5G networks.	5G	RSRP-based	TTT	➢ Bandwidth ➢ HO latency
[34]	Frequent HOs in millimeter-wave networks lead to significant signal attenuation and limited coverage.	5G	RL based on HO in SINR-based	TTT	➢ Throughput ➢ Handover rate
[35]	Frequent unnecessary HOs, network selection inefficiencies, and routing challenges due to high vehicle mobility.	LTE and mm-wave 5G	SNR-based and distance	Simulation time	➢ HO success ➢ HO failure ➢ Throughput ➢ Delay ➢ packet loss
[36]	Low network performance and frequent HO failures degrade communication performance in the IoV network, particularly in high-vehicle-density areas.	LTE and mm-wave 5G	RL based on HO for SNR-based	-	➢ Packet delivery ratio ➢ Packet loss ➢ Throughput ➢ HO failure
[37]	Addressing HO decision problems in dense HetNets to reduce energy consumption while maintaining excellent connectivity.	5G	Deep Q-learning-based HO in RSRP-based	Time slot duration (Ts)	➢ Throughput ➢ Energy consumption ➢ Handover rate

**Table 2:** Network parameters for simulation scenarios.

Network Parameters	Values
Network types	5G/6G network
Number of BSs	183
Network 1 carrier frequency GHz	28
Network 1 system bandwidth GHz	0.5
Network 2 carrier frequency GHz	120
Network 2 system bandwidth GHz	2
Cell radius	200
Antenna configuration	Omnidirectional antenna sectoring
Transmit power (dBm)	46
Path loss model	$\mathrm{PL}\;=\;40\;\times \;(1\;-\;4\;(10^{-3}$$)d)\log_{10}\left(R\right)$ $-\;18\;\times \;\log_{10}\left(d\right)$ $+\;21\;\times \;\log_{10}\left(f\right)$ + 80 dB
Antenna gain (dBi)	15
Number of UEs	5
Number of simulation cycles	20,000
gNB height (*hUE*) (m)	1.5
UE speed (Km/hr)	[40, 60, 80, 100, 120, 140]
Handover time execution	40 ms
UE max Tx power (dBm)	23
UE min Tx power (dBm)	−40
HOM (dB)	3
TTT (ms)	160
HO decision	RSRP and SINR; see the algorithm equations in (1) and (3)

**Table 3:** Simulation results for HO performance for all systems across mobile speed scenarios.

Average HO probability						
UE speed	40	60	80	100	120	140
System 1	0.1853	0.1869	0.1678	0.1467	0.1778	0.1936
System 2	0.6974	0.8243	0.6089	0.6763	0.6022	0.7110
System 3	0.3473	0.3513	0.3095	0.3026	0.3035	0.3113
System 4	0.6402	0.6458	0.6445	0.7101	0.6484	0.6425
Average HO ping-pong						
System 1	0.1692	0.1647	0.1466	0.1216	0.1427	0.1751
System 2	0.0001	0	0.0001	0	0	0
System 3	0.3111	0.3066	0.2529	0.2568	0.2523	0.2497
System 4	0.00001	0	0.00001	0	0	0
Average radio link failure						
System 1	0.0087	0.0101	0.0081	0.0070	0.0091	0.0108
System 2	0.0332	0.0460	0.0296	0.0338	0.0259	0.0392
System 3	0.0191	0.0188	0.0156	0.0152	0.0171	0.0160
System 4	0.0362	0.0348	0.0320	0.0341	0.0373	0.0337
Average data rate						
System 1	0.1195	0.0844	0.1718	0.1171	0.1638	0.1202
System 2	0.1941	0.1700	0.2361	0.1911	0.2065	0.1924
System 3	0.1880	0.1833	0.1833	0.1594	0.1774	0.1547
System 4	0.2217	0.2163	0.2054	0.1843	0.2025	0.1772

**Table 4:** Systems evaluation performance based on KPIs.

KPIs	System 1	System 2	System 3	System 4
RSRP	−74.6373	−62.4544	−80.2183	−73.7664
SINR	−3.1598	2.1798	−0.67715	2.0809
HOP	0.17636	0.68668	0.3209	0.6553
HOPP	0.1533	0.000047	0.2716	0.000023
RLF	0.00898	0.034633	0.0170	0.0347
Data rate	0.13127	0.20361	0.1771	0.2055

**Table 5:** Performance gain evaluation for all systems across the KPI with the best performance.

KPI	System 1 vs. System 2	System vs. System 4	Best System
RSRP	16.32	8.04	System 2 and System 4
SINR	168.99	407.31	System 2 and System 4
HOP	74.32	51.03	System 1 and System 3
HOPP	99.97	99.99	System 2 and System 4
RLF	74.07	51.01	System 1 and System 3
Data rate	55.11	16.04	System 2 and System 4

## Data Availability

The datasets presented in this article are not readily available because the data are part of an ongoing study. Requests to access the datasets should be communicated to the corresponding author.

## Abbreviations

The following abbreviations are used in this manuscript:

Abbreviations	Definition
5G	Fifth generation;
6G	Sixth generation;
AI	Artificial intelligence;
CNNs	Convolutional neural networks;
DDRL	Double deep reinforcement learning;
DL	Deep learning;
DNNs	Deep neural networks;
gNB	Next-generation node B;
HCP	Handover control parameter;
HO	Handover;
HOD	Handover decision;
HOM	Handover margin;
HOP	Handover probability;
HOPP	Handover ping-pong;
IoT	Internet of Things;
IoV	Internet of Vehicles;
k-NN	K-nearest neighbor;
KPI	Keys performance indicator;
LTE	Long-term evolution;
MEC	Mobile edge computing;
ML	Machine learning;
mmWave	Millimeter-wave;
MS	Mobile station;
MU	Mobile users;
QoE	Quality of experience;
QoS	Quality of service;
RL	Reinforcement learning;
RLF	Radio link failure;
RSRP	Reference signal receiver power;
TTT	Time to trigger;
Tbps	Terabits per second;
SINR	Signal-to-interference-plus-noise ratio.

## Appendix A

Algorithm A1 is presented in Section 4.6.

**Algorithm A1.** Handover Decision Algorithm						
*1.* Start						
***2. Initialize*** *network parameters and mobility model*						
***3. Initialize*** *the simulation cycles (t = 1…* T)						
*4. Select the input parameter: RSRP, SINR, and UEs speed*						
*5. Setting output: HOM and TTT values are fixed*						
*6. Evaluate the input parameters*						
*7. If* $RSRP_{Target\;}\geq \;RSRP_{Serving}$						
*8.*	TTT and HOM are fixed values					
*9.*		If T = 1 then				
*10.*			HO decision is false			
*11.*			***If*** $RSRP_{Target}\geq RSRP_{Serving}+HOM\;$			
*12.*				*If Timer trigger* ≥ *TTT then*		
*13.*					*HO decision is true*	
*14.*					*Initiate all three HO phases: preparation, execution, and completion.*	
*15.*				***else***		
*16.*					***HO’s decision is false***	
*17.*				***end***		
*18.*			***else***			
*19.*					***HO decision is false***	
*20.*			***end***			
*21.*		***else***				
*22.*			* Return to step 6 to evaluate the input based on SINR*			
*23.*		***If*** $SINR_{Target}\geq SINR_{Serving}$				
*24.*			TTT *and* HOM *are fixed values*			
*25.*			*If* T = 1 then			
*26.*				HO’s decision is false		
*27.*				***If*** $SINR_{Target}\geq SINR_{Serving}+HOM\;$		
*28.*					*If* Timer trigger ≥ TTT *then*	
*29.*						HO *decision is true*
*30.*						*Initiate all three HO phases:* *(preparation, execution, and completion.)*
*31.*					***else***	
*32.*						HO’s *decision is false*
*33.*					*end*	
*34.*				*else*		
*35.*						HO’s *decision is false*
*36.*				*end*		
*37.*			***else***			
*38.*		***else***				
*39.*	***end***					

## References

[1] Ismail L., Buyya R. (2022). Artificial intelligence applications and self-learning 6G networks for smart cities digital ecosystems: Taxonomy, challenges, and future directions. Sensors.

[2] Warrier A., Aljaburi L., Whitworth H., Al-Rubaye S., Tsourdos A. (2024). Future 6G communications powering vertical handover in non-terrestrial networks. IEEE Access.

[3] Sharma S., Popli R., Singh S., Chhabra G., Saini G.S., Singh M., Sandhu A., Sharma A., Kumar R. (2024). The role of 6G technologies in advancing smart city applications: Opportunities and challenges. Sustainability.

[4] Junejo Y.S., Shaikh F.K., Chowdhry B.S., Ejaz W. (2025). Adaptive handover management in high-mobility networks for smart cities. Computers.

[5] Karmakar R., Kaddoum G., Chattopadhyay S. (2022). Mobility management in 5G and beyond: A novel smart handover with adaptive time-to-trigger and hysteresis margin. IEEE Trans. Mob. Comput..

[6] Ullah Y., Roslee M.B., Mitani S.M., Khan S.A., Jusoh M.H. (2023). A survey on handover and mobility management in 5G HetNets: Current state, challenges, and future directions. Sensors.

[7] Tashan W., Shayea I., Aldirmaz-Colak S. (2024). Analysis of mobility robustness optimization in ultra-dense heterogeneous networks. Comput. Commun..

[8] Tashan W., Shayea I., Aldirmaz-Colak S., Ergen M., Azmi M.H., Alhammadi A. (2022). Mobility robustness optimization in future mobile heterogeneous networks: A survey. IEEE Access.

[9] Saad W.K., Shayea I., Alhammadi A., Sheikh M.M., El-Saleh A.A. (2023). Handover and load balancing self-optimization models in 5G mobile networks. Eng. Sci. Technol. Int. J..

[10] Alraih S., Nordin R., Shayea I., Abdullah N.F., Abu-Samah A., Alhammadi A.J.W.C., Computing M. (2022). Effectiveness of handover control parameters on handover performance in 5G and beyond mobile networks. Wirel. Commun. Mob. Comput..

[11] Bang J.-h., Oh S., Kang K., Cho Y.-J. (2019). A Bayesian regression based LTE-R handover decision algorithm for high-speed railway systems. IEEE Trans. Veh. Technol..

[12] Chen Y., Niu K., Wang Z. (2021). Adaptive handover algorithm for LTE-R system in high-speed railway scenario. IEEE Access.

[13] Saad W.K., Shayea I., Hamza B.J., Mohamad H., Daradkeh Y.I., Jabbar W.A. (2021). Handover parameters optimisation techniques in 5G networks. Sensors.

[14] Shayea I., Ergen M., Azizan A., Ismail M., Daradkeh Y.I. (2020). Individualistic dynamic handover parameter self-optimization algorithm for 5G networks based on automatic weight function. IEEE Access.

[15] Huang T., Yang W., Wu J., Ma J., Zhang X., Zhang D. (2019). A survey on green 6G network: Architecture and technologies. IEEE Access.

[16] David K., Berndt H. (2018). 6G vision and requirements: Is there any need for beyond 5G?. IEEE Veh. Technol. Mag..

[17] Akbar M.S., Hussain Z., Ikram M., Sheng Q.Z., Mukhopadhyay S.C. (2025). On challenges of sixth-generation (6G) wireless networks: A comprehensive survey of requirements, applications, and security issues. J. Netw. Comput. Appl..

[18] Jha A.V., Appasani B., Khan M.S., Zeadally S., Katib I. (2024). 6G for intelligent transportation systems: Standards, technologies, and challenges. Telecommun. Syst..

[19] Seaman E., Tejedor E., Kochhar R.K., Magnusson S., Stefan P. (2023). 6G Spectrum—Enabling the Future Mobile Life Beyond 2030.

[20] Udayananda G., Shyalika C., Kumara P. (2022). Rice plant disease diagnosing using machine learning techniques: A comprehensive review. SN Appl. Sci..

[21] Ferretti S., Ghini V., Panzieri F. (2016). A survey on handover management in mobility architectures. Comput. Netw..

[22] Park S., Kim J. Trends in LEO satellite handover algorithms. Proceedings of the 2021 Twelfth International Conference on Ubiquitous and Future Networks (ICUFN).

[23] Al-Quraan M., Khan A., Mohjazi L., Centeno A., Zoha A., Imran M.A. (2022). Intelligent blockage prediction and proactive handover for seamless connectivity in vision-aided 5G/6G UDNs. arXiv.

[24] Loutfi S.I., Shayea I., Tureli U., El-Saleh A.A., Tashan W., Caglar R. (2025). Machine learning for handover decision with mobile edge computing in 6G mobile network: A survey. Eng. Sci. Technol. Int. J..

[25] TelecomTrainer (2024). Handover in 5G. https://www.telecomtrainer.com/handover-5g/.

[26] Mahamod U., Mohamad H., Shayea I., Othman M., Asuhaimi F.A. (2023). Handover parameter for self-optimisation in 6G mobile networks: A survey. Alex. Eng. J..

[27] 3GPP (2022). NR; Radio Resource Control (RRC); Protocol Specification. https://portal.3gpp.org/desktopmodules/Specifications/SpecificationDetails.aspx?specificationId=3197.

[28] Kustiawan I., Liu C.-Y., Hsu D.F. (2017). Vertical handoff decision using fuzzification and combinatorial fusion. IEEE Commun. Lett..

[29] Rizkallah J., Akkari N. SDN-based vertical handover decision scheme for 5G networks. Proceedings of the 2018 IEEE Middle East and North Africa Communications Conference (MENACOMM).

[30] Song Y., Lim S.H., Jeon S.-W. Distributed online handover decisions for energy efficiency in dense HetNets. Proceedings of the GLOBECOM 2020—2020 IEEE Global Communications Conference.

[31] Satapathy P., Mahapatro J. (2022). An efficient multicriteria-based vertical handover decision-making algorithm for heterogeneous networks. Trans. Emerg. Telecommun. Technol..

[32] Satapathy P., Mahapatro J. (2023). An adaptive context-aware vertical handover decision algorithm for heterogeneous networks. Comput. Commun..

[33] Silva F.S.D., Schneider L.M., Rosário D., Neto A.V. Network slicing mobility aware control to assist handover decisions on e-health 5g use cases. Proceedings of the 2022 International Wireless Communications and Mobile Computing (IWCMC).

[34] Mollel M.S., Abubakar A.I., Ozturk M., Kaijage S., Kisangiri M., Zoha A., Imran M.A., Abbasi Q.H. (2020). Intelligent handover decision scheme using double deep reinforcement learning. Phys. Commun..

[35] Hussain S.M., Yusof K.M., Yusof K. (2021). Dynamic Q-learning and fuzzy CNN based vertical handover decision for integration of DSRC, mmwave 5G and LTE in internet of vehicles (IoV). J. Commun..

[36] Pramod Kumar P., Sagar K. (2023). Reinforcement learning and neuro-fuzzy GNN-based vertical handover decision on internet of vehicles. Concurr. Comput. Pract. Exp..

[37] Song Y., Lim S.H., Jeon S.-W. (2023). Handover Decision Making for Dense HetNets: A Reinforcement Learning Approach. IEEE Access.

[38] 3GPP Study on Channel Model for Frequencies from 0.5 to 100 GHz. https://portal.3gpp.org/desktopmodules/Specifications/SpecificationDetails.aspx?specificationId=3173.

[39] 3GPP (2025). Evolved Universal Terrestrial Radio Access (E-UTRA); Radio Frequency (RF) System Scenarios. https://portal.3gpp.org/desktopmodules/Specifications/SpecificationDetails.aspx?specificationId=2592.

[40] Gures E., Shayea I., Sheikh M., Ergen M., El-Saleh A.A. (2023). Adaptive cell selection algorithm for balancing cell loads in 5G heterogeneous networks. Alex. Eng. J..

